# Advanced cardiovascular risk prediction in the emergency department: updating a clinical prediction model – a large database study protocol

**DOI:** 10.1186/s41512-021-00105-7

**Published:** 2021-10-07

**Authors:** Charles Reynard, Glen P. Martin, Evangelos Kontopantelis, David A. Jenkins, Anthony Heagerty, Brian McMillan, Anisa Jafar, Rajendar Garlapati, Richard Body

**Affiliations:** 1 Division of Cardiovascular Sciences, University of Manchester, Manchester, UK; 2 Emergency Department, Manchester University NHS Foundation Trust, Manchester, UK; 3 Division of Informatics, Imaging and Data Science, Faculty of Biology, Medicine and Health, University of Manchester, Manchester Academic Health Science Centre, Manchester, UK; 4 Centre for Primary Care and Health Services Research Division of Population Health, Health Services Research and Primary Care School of Health Sciences Faculty of Biology, Medicine and Health University of Manchestern, Manchester, UK; 5 Humanitarian and Conflict Response Institute, University of Manchester, Manchester, UK; 6 Emergency Department, Royal Blackburn Hospital, East Lancashire Hospitals NHS Trust, Burnley, UK

**Keywords:** Clinical prediction model, Acute myocardial infarction, Calibration drift

## Abstract

**Background:**

Patients presenting with chest pain represent a large proportion of attendances to emergency departments. In these patients clinicians often consider the diagnosis of acute myocardial infarction (AMI), the timely recognition and treatment of which is clinically important. Clinical prediction models (CPMs) have been used to enhance early diagnosis of AMI. The Troponin-only Manchester Acute Coronary Syndromes (T-MACS) decision aid is currently in clinical use across Greater Manchester. CPMs have been shown to deteriorate over time through calibration drift. We aim to assess potential calibration drift with T-MACS and compare methods for updating the model.

**Methods:**

We will use routinely collected electronic data from patients who were treated using TMACS at two large NHS hospitals. This is estimated to include approximately 14,000 patient episodes spanning June 2016 to October 2020. The primary outcome of acute myocardial infarction will be sourced from NHS Digital’s admitted patient care dataset. We will assess the calibration drift of the existing model and the benefit of updating the CPM by model recalibration, model extension and dynamic updating. These models will be validated by bootstrapping and one step ahead prequential testing. We will evaluate predictive performance using calibrations plots and c-statistics. We will also examine the reclassification of predicted probability with the updated TMACS model.

**Discussion:**

CPMs are widely used in modern medicine, but are vulnerable to deteriorating calibration over time. Ongoing refinement using routinely collected electronic data will inevitably be more efficient than deriving and validating new models. In this analysis we will seek to exemplify methods for updating CPMs to protect the initial investment of time and effort. If successful, the updating methods could be used to continually refine the algorithm used within TMACS, maintaining or even improving predictive performance over time.

**Trial registration:**

ISRCTN number: ISRCTN41008456

## Background

Chest pain accounts for approximately 6% of all Emergency Department (ED) attendances [1]. Despite recent advances in diagnostic technology and changes to national guidelines [2, 3], it remains the most common reason for emergency hospital admission in England and Wales [1]. These patients are frequently admitted to undergo diagnostic evaluation for suspected acute coronary syndrome (ACS). Improved diagnostic pathways could allow those without an ACS diagnosis (over 100,000 patients per year in England and Wales) to be discharged from the ED without an unnecessary hospital admission. Equally it is integral that we try to capture as many ACS diagnoses as we can, since a missed ACS infers twice the mortality of a detected ACS [4].

The Troponin-only Manchester Acute Coronary Syndromes (T-MACS) decision aid can be used to rapidly rule in, rule out and risk stratify patients with suspected ACS [5]. T-MACS was derived by logistic regression, using details of a patient's symptoms with electrocardiographic (ECG) findings and cardiac troponin (cTn) concentrations, measured on arrival at ED, to calculate the probability that a patient has ACS. T-MACS classified patients with <2% probability of ACS as being 'very low risk', in this population this strategy identified 40% of patients as eligible for safe, immediate discharge from the ED [5].

T-MACS has been externally validated in 1,459 patients from three prospective studies in the United Kingdom [5], 1,244 patients from Australasia [6], and multi-centre prospective trials from the United Kingdom [7], Thailand [8] and Norway [9], each of which demonstrated acceptable predictive performance. A pilot randomized controlled trial of a precursor version of the algorithm showed that its use led to significantly more safe discharges from the ED within 4 hours of arrival than standard care [10]. The data from UK studies (which did not rely on the use of surrogate variables) consistently show that over 40% of patients are categorised as very low risk and can have ACS ‘ruled out’ with one blood test. It has been shown to safely reduce unnecessary hospital admissions, outperforming the algorithms currently advocated in NICE guidelines [2, 11].

### Countering calibration drift

However, the performance (calibration and discrimination) of many clinical prediction models, such as T-MACS, is likely to decline with time [12, 13]. For example, this has been demonstrated previously with the EuroScore model that predicts short-term mortality after cardiac surgery [14]. Therefore, the same phenomenon is likely to occur with the T-MACS algorithm as patient demographics change and diagnostic technology evolves. Indeed, the very fact that T-MACS is implemented in practice can lead to it losing diagnostic performance, since the implementation of the model changes the predictor-outcome associations and the case-mix, meaning that the performance of the model degrades over time [15, 16].

In part, the above issues with “calibration drift” can be attributed to the fact the algorithm itself is static, having been derived in one sample over a fixed time-period. It is unlikely to be the optimal algorithm for early diagnosis in various locations with diverse populations, due to the population and, possibly, intervention heterogeneity. This has been attempted previously with the EuroScore, which was shown to demonstrate calibration drift due to changing demographics [14]. Siregar et al investigated the merits of various methods through which to update such models [17]. They found many had a similar improvement on the clinical prediction models (regression co-efficient updating and dynamic updating).

### Model updating and dynamic approaches to clinical prediction models

Statistical methods have previously been proposed to overcome issues such as calibration drift, by allowing prediction models to be re-derived and validated to maintain their predictive performance through time [18]. Such cycles of learning allow the models to account for demographic shifts and changes in diagnostic technology. This has several advantages over continuously re-developing the model de novo, as model updating utilises existing evidence (current versions of the model) and can potentially be delivered in almost real-time. Specifically, several different methods for updating clinical prediction models have been suggested [12, 18]; including regression coefficient updating, meta-model updating and dynamic updating. Regression coefficient updating only modifies individual coefficients within the model from a singular further analysis. Bayesian dynamic updating allows for continuous updating and derivation, once the method has been evaluated it can theoretically continuously re-derive with new data [19, 20]. Siregar et al’s analysis of dynamic updating suggested that a Bayesian approach may yield greater improvements in accuracy, when the sample is small [17]. Strobl et al [21] demonstrated that in updating prostate cancer risk assessment tools, there were also multiple methods that yielded similar improvement, with the exception of Random Forest regression (a machine learning form of dynamic updating) which was substantially worse than others.

In summary, T-MACS requires protection against calibration drift, and as such we aim to utilise prediction model updating methods to recalibrate it through time.

Here, we describe the protocol for the study that will deliver these objectives, in full accordance with the Transparent Reporting of the Predictive accuracy of a multivariable prediction model for Individual Prognosis Or Diagnosis (TRIPOD) guidelines [22].

## Methods

### Study arm design and study setting

This will be a multi-centre retrospective cohort study. The study will use data collected from emergency departments at Manchester Royal Infirmary (MRI), Royal Blackburn Teaching Hospital (RBTH) and Burnley General Teaching Hospital (BGTH). MRI is a major trauma centre with 1,721 beds and an emergency department attendance of 104,449 in 2020, RBTH has 700 inpatient beds with an emergency department attendance of 104,009 in 2020 and BGTH has 219 beds and its urgent care centre has an annual attendance of 44,519. Each of these hospitals has implemented TMACS to guide the care of patients with suspected ACS.

### Study population

We will include patients who presented to the emergency department with chest pain and were assessed using the TMACS pathway since implementation at MRI, RBTH and BGTH. This is estimated to include approximately 14,000 patient episodes from June 2016 to October 2020.

### Sample Size

We utilised the sample size calculation described by Riley et al [23] and also the rule of 10 primary outcome cases by variable used in similar logistic regression analyses. TMACS includes seven variables. As it is also planned to incorporate time, geographical location and the outcome of two alternative clinical prediction models (adding 8 variables), it is anticipated that the analysis will require a minimum of 170 cases in the training/optimization set. The prevalence of the primary outcome is 6.9% in the first 1,033 patients treated with TMACS. Based on that prevalence, a minimum of 2,464 patients would be required. This sample size was larger than that calculated by Riley et al, so we opted to be conservative and use the higher initial calculation [23].

### Data Collection

This cohort will include patients who received routine care guided by TMACS, and whose data have been saved using bespoke interfaces deployed at Manchester Royal Infirmary (MRI), Royal Blackburn Teaching Hospital (RBTH) and Burnley General Teaching Hospital (BGTH). These tools are used in clinical practice and prospectively capture the data inputted by clinicians. This will be collated with data from local hospital servers to include: serial troponin assay results, and local diagnostic codes. This data will be cross-referenced with NHS Digital’s Hospital Episode Statistics (HES) database to include any diagnosis or intervention that occurs within 30 days of the index presentation. We will also link with the civil registry database for mortality outcomes.

### Data Validation

Assuring the quality of the data is vital for the integrity of this study, particularly as we are collating multiple databases across multiple organisations. We will use the principles laid out by Weiskopf et al to assure the quality of the data [24] (see Table 1).

**Table 1:** Data validation procedures adapted from Weiskopf et al [24]

Data Assessment Method	Data Quality Dimension	Databases	Data points
Gold Standard Comparison	Completeness & correctness	intra-EPR	laboratory results
		intra-EPR	personal identifiers
		intra-EPR	dates of events
Data element agreement	Completeness, correctness, concordance	intra-EPR	physiological parameters
		intra-EPR	dates of events
Element presence	Completeness	NHSD & EPR	All data points
Data source agreement	Completeness, concordance, plausibility	NHSD vs EPR	ICD-10 codes^b^
		NHSD vs EPR	dates of events
		EPR & paper records	CPM parameters^a^
Distribution comparison	Completeness, concordance, plausibility	EPR	Demographic data
		EPR	CPM covariates
		NHSD & EPR	outcome data
Validity check	Correctness and plausibility	intra-EPR	Demographic data
		intra-EPR	CPM covariate data
Log review	Correctness and currency	intra-EPR	CPM entry date
		NHSD	episode entry date

EPR – local electronic patient record, NHSD – National Health Service Digital (UK), CPM – clinical prediction model, ICD-10 – international classification of disease version 10. ^a^ this is detailed in the supplementary appendix ^b^ a sensitivity analysis is also planned, see the outcome variable subsection

### Outcome Variables

The primary outcome will be acute myocardial infarction (AMI) within 30 days. Patients will be considered to have a diagnosis of AMI if they have either a coded clinical diagnosis of AMI locally or held centrally with NHS Digital. Only primary ICD-10 codes will be used for the outcome, however a sensitivity analysis will be conducted where a code at any position is used. The relevant ICD-10 codes are: I21, I22, or I23 [25].. A secondary composite outcome of major adverse cardiovascular events within 30 days will also be measured including acute myocardial infarction, death (as per civil registry) and revascularisation. ICD-10 outcomes include I21, I22, I23, I46, R96, R99, K40-50, K63, and K75

The use of coded diagnoses is essential for the process to be automated in future. However, the effect of accepting these definitions must be carefully considered due to concerns over the limitations of the coding databases both centrally and locally. This will be explored by conducting data validation and the effect of the differing outcomes will be examined in sensitivity analyses. We will blind the adjudicators to the TMACS inputs and prediction.

In data validation we will examine the local coded diagnoses of any patients who had at least one cardiac concentration above the 99^th^ percentile upper reference limit for the assay and an absolute change of at least half the 99th percentile on serial sampling (for samples drawn 3-6 hours apart). We will not examine patients with two (adequately timed) troponin concentrations within the normal range as they cannot fulfil the diagnosis of AMI. We will adjudicate outcomes by a central committee. AMI will be defined in accordance with the universal definition of myocardial infarction, which requires a rise and/or fall of cardiac troponin with at least one concentration above the 99th percentile upper reference limit of the assay. In addition, patients must have at least one of: symptoms compatible with myocardial ischaemia, ECG changes compatible with ischaemia, imaging evidence of new loss of viable myocardium or identification of intracoronary thrombus at coronary angiography. In the GM-TMACS project, initial implementation of TMACS will require all patients to have two cardiac troponin tests drawn 3 hours apart. Thus, all patients included in the analyses presented here will have an acceptable reference standard for the diagnosis of AMI according to national and international guidelines [2, 26]. Diagnoses will be adjudicated by two independent investigators with reference to all relevant clinical investigations. Disagreements will be resolved by consulting with a third independent investigator.

### Analysis

The methodology optimising predictive performance for updating the TMACS algorithm will be identified from four candidate types. Predictive performance will be assessed by calibration plots, Brier scores, discrimination will be assessed with the c-statistic compared with DeLong’s method [27]. We will examine continuation of the current model (status quo), model recalibration, model revision, and Bayesian dynamic modelling [13, 18]. TMACS currently returns a probability of ACS, which is then used to classify patients into a categorical risk group (Eq 1 ). We will examine the re-classification of patients from the original TMACS algorithm and the dynamic modelling approach [28]. We will calculate the observed risk of the reclassified cases.

1 $$ l={\mathit{\log}}_b\frac{p}{1-p}=1.713{x}_e+0.847{x}_a+0.607{x}_r+1.417{x}_v+2.058{x}_s+1.208{x}_h+0.089{x}_t-4.766 $$

Equation [1] - The TMACS clinical prediction model. *l* = log-odds of the primary outcome acute myocardial infarction, *x*_*e*_ = presence of ECG ischaemia, *x*_*a*_ = crescendo angina, *x*_*r*_ = paint radiating to the right arm, *x*_*v*_= pain associated with vomiting, *x*_*s*_ = sweating observed, *x*_*h*_= hypotension, and *x*_*t*_ = is high sensitivity troponin T result on arrival.

### Status Quo

The current iteration of the TMACS algorithm will be validated with the existing co-efficient and intercept (from the derivation study). This will serve as a baseline for comparison, and we will use it to assess for evidence of change of discrimination and calibration over time (Eq 2).

2 $$ {Z}_{sq}={\alpha}_{TMACS}+\sum \limits_{i\epsilon 1,\dots, 7}{\beta}_{i, TMACS}{x}_i $$

Equation 2: The current iteration of the TMACS algorithm, where *Z*_*sq*_ - the linear prediction of the current model, *α*_*TMACS*_ - intercept and *β*_*i*, *TMACS*_ previously derived regression coefficients.

### Model recalibration

In this method we will recalibrate the TMACS algorithm with the entire dataset and apply an overall weight to the original algorithm and derive a new intercept, this is described in Eq. 3 [29]. This has been included as it is the simplest and has been used previously to updated CPMs [30].

3 $$ {\displaystyle \begin{array}{c}{Z}_{mr}=\hat{\alpha}+{\hat{\beta}}_o{Z}_{sq}\\ {}{Z}_{mr}=\hat{\alpha}+{\hat{\beta}}_o{\alpha}_{TMACS}+\sum \limits_{i\in 1,\dots, 7}{\hat{\beta}}_o\ast \left({\beta}_{i, TMACS}{x}_i\right)\end{array}} $$

Equation 3: *Z*_*mr*_ - model updated by recalibration, $\hat{\alpha}$ is the re-estimated intercept, $\hat{\beta_o}$the new overall calibration slope, and *Z*_*sq*_ – is the linear prediction of the TMACS model.

### Model extension

Additional variables will be considered for incorporation from other clinical prediction models that have been used for the same purpose (Eq. 4). These include predictors from the HEART score and Thrombolysis in Myocardial Infarction (TIMI) risk score [29, 30]. We will re-derive the algorithm with these covariates to investigate any improvement in diagnostic characteristics [18, 29]..

4 $$ {Z}_e=\hat{\alpha}+\sum \limits_{i\epsilon 1,\dots, 7}{\beta}_i{x}_i+\sum \limits_{j\epsilon s}{\hat{\beta}}_j{x}_j $$

Equation 4: *Z*_*e*_ - model updated by extension, *β*_*i*_ are the original coefficients for the original covariables, s is the new covariates and ${\hat{\beta}}_i$ their new coefficients.

### Bayesian dynamic updating

Dynamic updating allows the original model’s intercepts and co-efficients to be updated after each patient episode, stabilising calibration and improving performance [12, 20]. This will be deployed incorporating guidance from our patient and public representatives. The representatives stated that if such a method were used then they felt that it initially ought to require human oversight. As such the initial updating will not be after each recorded patient episode instead it will be every three months for the first year to simulate a probationary period with quarterly meetings. After this period we will update the model after every patient episode. This is achieved through recursive estimation using the prediction equation

$$ {\beta}_t\mid {Y}^{t-1}\sim N\left(\ {\hat{\beta}}_{t-1},{R}_t\right) $$

Where *β* is a dimensional vector of regression coefficients, *Y*^*t* − 1^ is a set of past outcomes, t is a given time and $$ {R}_t=\raisebox{1ex}{${\hat{\sum}}_{t-1}$}\!\left/ \!\raisebox{-1ex}{${\lambda}_t$}\right. $$. *λ*_*t*_ is known as the forgetting factor, which down-weights past observations by inflating the variance, and will be chosen in order to enable the sample size to continue to meet the specifications laid out by Riley et al [23].

When this is then taken into a Bayesian framework, the posterior is proportional to the product of the probability distribution at time t and t-1, giving

$$ p\left({\beta}_t|{Y}^t\right)\propto p\Big({y}_t\left|\ {\beta}_t\right)p\left({\beta}_t|{Y}^{t-1}\right)\propto likelihood\ x\  prior $$

### Validation

Model recalibration and model revision methods will be internally validated by using a bootstrap validation of 1000 samples. The dynamic updating methodologies will be internally validated with one-step a head prequential testing [13].

### Ethics and dissemination:

This study has received ethical approval from a research ethics committee and the confidentiality advisory group (references 19/WA/0311, and 19/CAG/0209).

The study is registered on the ISRCTN number: ISRCTN41008456

First, we aim to publish our findings in peer reviewed journals. The primary target audience for the clinical study will be emergency medicine physicians, acute medicine physicians, cardiologists, clinical biochemists, public health professionals and industry leaders in acute diagnostics.

Further we will aim to present our findings at international and national conferences with relevant target audiences (e.g. European Society for Emergency Medicine Annual Congress, European Society of Cardiology Annual Conference, Royal College of Emergency Medicine Annual Scientific Conference). In addition, we will develop a public engagement strategy in conjunction with Public Programmes and our patient groups, in order that the local population have the opportunity to learn about our work and to engage with future work.

## Discussion

We aim to recalibrate TMACS protecting the research investment of time and money, but potentially also improving it’s clinically efficacy. However, this method could be applied to any clinical prediction model, a plethora of which are deployed within emergency medicine. These range from the Well’s score for deep vein thrombosis to the Ottawa ankle score for fractures [31, 32]. These were all derived and then externally validated, but subsequently their upkeep stopped.

The recent focus of research has been the development and deployment of new clinical prediction models. Here we present a method that follows the paradigm shift in the focus of modelling research. The scientific community must adapt to an overly saturated environment of clinical prediction models [33, 34], part of the answer is assessing what already exists and seeking to protect and improve it . Not only is this an efficiency but it also recalibrates the community of clinical modelers to follow one of the central thesis of science, to build on the work of others [35].

## Data Availability

Due to data governance restrictions from the confidentiality advisory group and NHS Digital the sharing of data is not possible. However, requests to collaborate are welcome.
